# Supplementary material for: Structure and electrochromism of two-dimensional octahedral molecular sieve h’-WO3
Source: Nat Commun. 2019 Jan 18;10:327. doi: 10.1038/s41467-018-07774-x (PMC6338762; doi:10.1038/s41467-018-07774-x)
Supplement: Supplementary file 1 — Supplementary Information [file 41467_2018_7774_MOESM1_ESM.pdf]

Supplementary Information

Structure and electrochromism of two-dimensional octahedral molecular sieve h'-WO_3

Julie Besnardiere,[1,#] Binghua Ma,[1,#] Almudena Torres-Pardo,[2] Gilles Wallez,[3] Houria Kabbour,[4] José M. González-Calbet,[2,5] Hans Jürgen Von Bardeleben,[6] Benoit Fleury,[7] Valérie Buissette,[8] Clément Sanchez,[1] Thierry Le Mercier,[8] Sophie Cassaignon,[1] David Portehault[1*]

[1] Sorbonne Université, CNRS, Collège de France, PSL Research University, Laboratoire Chimie de la Matière Condensée de Paris, LCMCP, 4 Place Jussieu, F-75005 Paris, France.

[2] Departamento de Química Inorgánica, Facultad de Químicas, Universidad Complutense, 28040-Madrid, Spain

[3] PSL Research University, Chimie ParisTech, CNRS, Institut de Recherche de Chimie de Paris, 11 rue Pierre et Marie Curie, 75005 Paris, France

[4] Univ. Lille, CNRS, ENSCL, Centrale Lille, Univ. Artois, UMR 8181-UCCS-Unité de Catalyse et de Chimie du Solide, F-59000 Lille, France

[5] Centro Nacional de Microscopía Electrónica, Universidad Complutense, 28040 Madrid, Spain

[6] Sorbonne Université, CNRS, Institut des Nanosciences de Paris, INSP, 4 Place Jussieu, F-75005 Paris, France

[7] Sorbonne Université, CNRS, Institut Parisien de Chimie Moléculaire, IPCM, F-75005 Paris, France

[8] RHODIA-Solvay, Centre de Recherches d'Aubervilliers, 52 rue de la Haie-Coq, 93308 Aubervilliers Cedex (France)

[#] These authors contributed equally

Corresponding author: david.portehault@upmc.fr

Supplementary Discussion………………………………………………….… Page 3
Structure resolution……………………………………………………… Page 3
Table 1. Structure resolution parameters…………………………..…… Page 4
Additional nanostructure characterization……………………………..…. Page 5
Figure 1. Size distributions…………………………………………….. Page 5
Figure 2. Powder XRD pattern of the as-obtained product……............ Page 5
Figure 3. UV-visible-near IR spectrum and its analysis….……………. Page 6
Electron Spin Resonance (ESR) and Ferromagnetic Resonance (FMR)..... Page 6
Figure 4. X-band ESR spectrum of slightly reduced **h'-WO_3** at 4 K…… Page 7
Figure 5. X-band ESR spectra of slightly reduced **h'-WO_3** at 4 K before and under photoexcitation ….…………………………. Page 7
Figure 6. X-band and Q-band FMR spectra of **h'-$H_{0.07}WO_3$** at room T… Page 9
Figure 7. X-band FMR/ESR spectra of **h'-$H_{0.07}WO_3$** as a function of T under photoexcitation. …………………...…………………. Page 9
Figure 8. ESR spectrum at 4 K of a Mo^{4+}-X center in **h'-$H_{0.07}WO_3$**…… Page 10
Composition of h'-WO_3 and its hydrogen bronze……………………...... Page 11
Figure 9. EDS spectrum of the h' bronze ……………………………… Page 12
Figure 10. W4f XPS area for the h' bronze and h-WO_3………...…… Page 12
Figure 11. STEM-ABF image of the **h'-$H_{0.07}WO_3$** bronze……..……… Page 13
Modeling …………….………….………………………….…………..….….. Page 14
Figure 12. Total DOS of the oxidized sample…………..…………..… Page 14
Figure 13. Scheme and DOS calculated for the H inserted h' phase, H in the center of the $(WO_6)_6$ channels. ………………………. Page 15
Figure 14. Scheme and DOS calculated for the H inserted h' phase, H forming hydroxo groups in the $(WO_6)_6$ channels...……..….. Page 16
Specific surface area………………….…………………………….…….. Page 17
Figure 15. N_2 sorption isotherms at 77K……………...……………… Page 17
Electrochromic properties…………………………………………………..Page 18
Figure 16. SEM image of the thickness of a **h'-WO_3**/FTO film.……..… Page 18
Figure 17. **h-WO_3** nanorods used as reference for electrochromism…... Page 19
Figure 18. Cyclic voltammograms of **h'-WO_3** and **h-WO_3** electrodes..... Page 20
Figure 19. UV-visible spectra of colored and bleached **h'-WO_3** films.... Page 20
Figure 20. Long term cycling of a **h'-WO_3** film on an FTO susbtrate….. Page 21
Figure 21. Long term cycling of a **h'-WO_3** film on an FTO susbtrate...... Page 22
Supplementary References…………………………………………….…. Page 23

Supplementary Discussion

Structure resolution

A hexagonal cell was defined on the basis of the HRTEM pictures of the (001) plane, and taking the thickness of one octahedra layer as the *c*-parameter. These parameters allowed a satisfactory indexing of all the XRD peaks, except those at $2\theta = 44.60$ and 49.17 ° that were ascribed to an impurity. The Rietveld refinement, performed in Le Bail's (profile matching) mode with help of the Fullprof suite[1] confirmed this hypothesis. A satisfactory model of the XRD pattern was obtained using the Thompson-Cox-Hastings profile function with anisotropic Scherrer's broadening to take into account the platelet shape of the crystallites. Besides, the uniaxial strain model was implemented because of the presence of lattice defects as will be shown below. To avoid a mix-up with the previously known hexagonal h-WO_3 form,[2] the present one is termed **h'-WO_3**.

The Patterson (001) maps synthesized in the *P*6 space group allowed to locate the tungsten atoms and confirmed the array observed by HRTEM. Then, the oxygen atoms were spotted from the Fourier maps. All the atomic coordinates were found to be fully compatible with the special positions of S. G. *P*6/*mmm*. *A posteriori* refinements performed in the *P*6/*m* and *P*6*mm* subgroups turned out to yield similar atomic positions without improving significantly the reliability factors.

The refinement of this set of atomic positions and thermal factors yielded nevertheless a poorly satisfactory model ($R_{\mathrm{Bragg}} = 0.038$; $\chi^2 = 31$), because of strong residuals in electron density in the cations (001) plane, matching with the positions of irregular tungsten atoms spotted by HRTEM. So a second set of atoms was introduced in the refinement, termed prime, in order to refine the crystal structure as a ((1-*x*)WO_3 + *x*W'O'$_3$) solid solution, with *x* standing for the occupancy rate of the irregular atoms. Because of the faint weight of the O' atoms, soft constraints based upon the W-O distances had to be applied to the W'-O' homologues during the refinement. The final reliability factors for the "faulted" model were clearly more satisfactory than those of the perfect one. The main crystallographic data are summarized in **Supplementary Table 1**. For more details, see the Supplementary Crystallographic Information File (CIF).

Supplementary Table 1. Main acquisition, refinement and lattice data for **h'-WO**$_3$. Corresponding atomic positions, thermal and occupancy factors.

apparatus	PanAlytical X'Pert Pro	atom	position	*x*	*z*	*B*** (Å^2)	occupancy
anode, monochromator	CuKα (40 kV, 45 mA), Ge (111)	W	6*m*	0.21180(3)	1/2	2.43(1)	0.8578(5)
scan range, step, time	$8.00 \leq 2\theta \leq 130.00$ °, 0.013 °, 12 h	O1	6*l*	0.2186(3)	0	0.41(5)	"
measured reflections	173	O2	6*k*	0.2534(5)	1/2	"	"
intensity / profile parameters	14 / 10	O3	6*m*	0.4188(5)	1/2	"	"
reliability factors	R_P = 0.023; R_{WP} = 0.032; R_{Bragg} = 0.020; χ^2 = 8.3	W'	6*k*	0.3559(2)	1/2	2.43(1)	0.1422(5)
system, space group	hexagonal, *P*6/*mmm* (191)	O1'	6*j*	0.335(2)	0	0.41(5)	"
cell parameters *, volume	*a* = 9.9975(8) Å; *c* = 3.9199(6) Å; *V* = 339.3(1) Å^3	O2'	6*m*	0.144(2)	1/2	"	"
formula per cell / calc. density	6 / 6.81	O3'	6*m*	0.423(1)	1/2	"	"

* esd's on *a* and *c* given by Fullprof were increased by a 10 factor. **B_{iso} for O's; B_{eq} for W's (B_{11} = 0.48(1) Å^2; B_{33} = 6.33(3) Å^2; B_{12} = 0.35(2) Å^2)

cation-oxygen bond lengths: W-O1 : 1.963(1) Å; W-O2: 1.943(7) Å; W-O3: 1.874(5) Å

W'-O1': 1.972(4) Å; W'-O2': 1.88(3) Å; W'-O3': 1.89(1) Å

Comments on the CheckCIF report:

- Level B alerts concern the unusual high U_{33}/U_{11} = 13 ratio for W and W', that could result from either a possible splitting of these atoms on both sides of the (001) mirror plane (increasing U_{33}), or to the disorder in the same plane, resulting in overlapping atomic positions.

- Level G alerts warn about the (real) structural disorder, the use of soft constraints on the W'-O' distances and some minor discrepancies between the imaginary parts of the scattering factors used by Fullprof and those recommended by the IUCr.

Additional nanostructure characterization

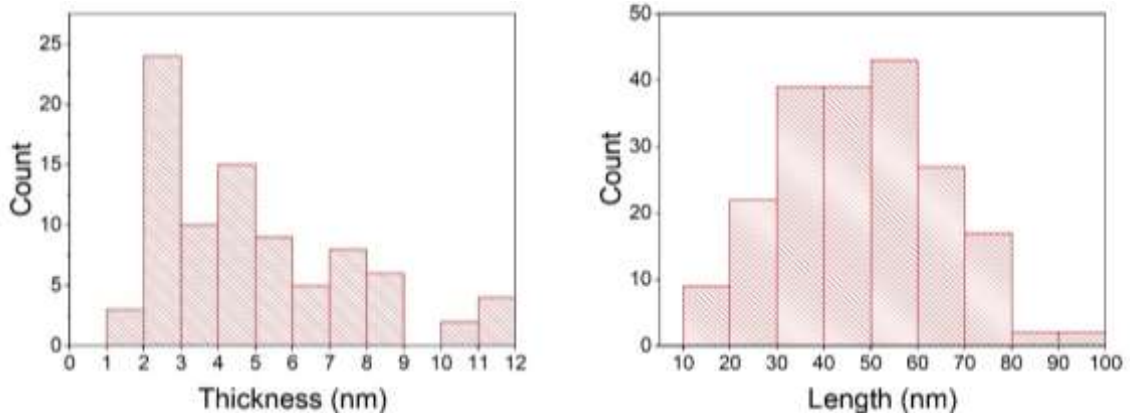

Supplementary Figure 1. Size distributions of nanoplatelets: (left) thickness and (right) basal face length.

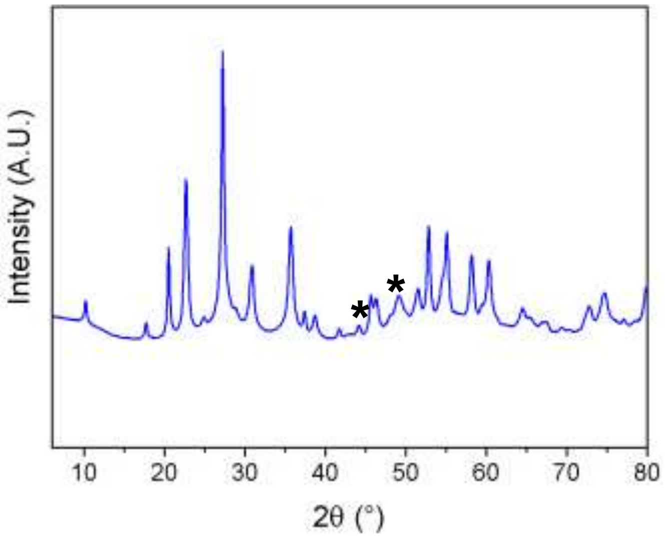

Supplementary Figure 2. XRD pattern of the synthesized powder. Stars highlight two peaks (2θ = 44.60 and 49.17°) attributed to an impurity, possibly a tungsten oxide hydrate.

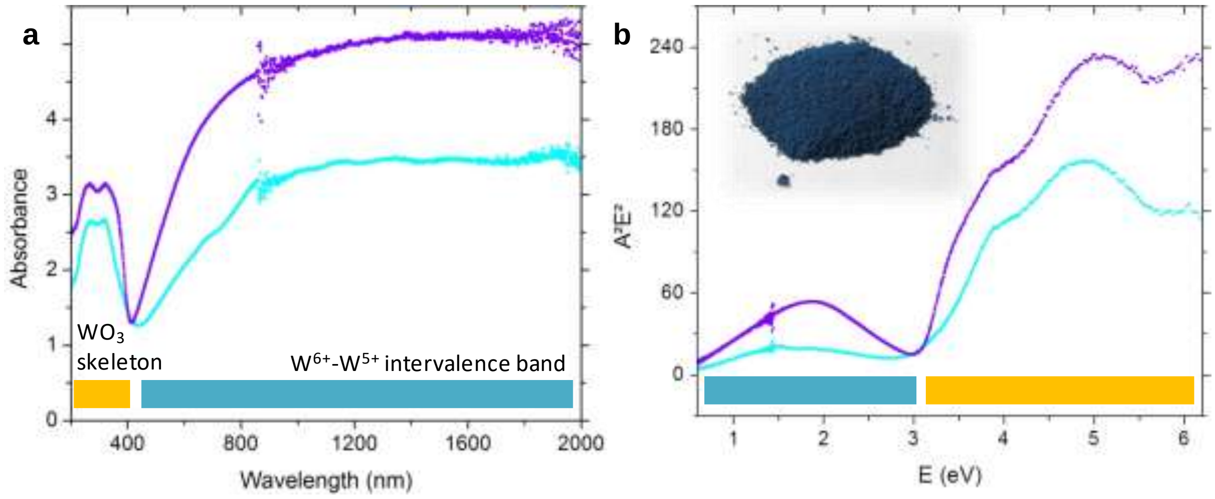

Supplementary Figure 3. (**a**) UV-visible-near IR spectrum of the **h'** bronze. For the sake of comparison, the spectrum of an ammonium bronze of **h-WO3** is also presented (blue curves). (**b**) A^2E^2=f(E) representation, used to evaluate the band gap with the hypothesis of a direct gap, usually observed for tungsten oxides. The inset shows a batch of **h'-WO3** bronze powder in actual colors.

Electron Spin Resonance (ESR) and Ferromagnetic Resonance (FMR)

Slightly reduced **h'-WO3**. The EPR spectrum at 4 K of **h'-WO3** slightly reduced upon exposure to air and light is shown in **Supplementary Figure 4**. The sample shows two distinct signals (ESR1, ESR2), which correspond to systems of diluted paramagnetic centers. The first signal ESR1 is characterized by an axial g-tensor with values of $g_{//}$ = 1.899 and g_L = 1.835. The second signal ESR2 is isotropic with a g factor of 1.641 and a linewidth of 150 G. Both spectra are attributed to a spin S = 1/2 shallow donor and a tungsten W^{5+} ($5d^1$) S=1/2 defect respectively.[3–6] The anisotropy of the first signal ESR1 is characteristic of an axially distorted oxygen ligand field, with the main axis of the **g** tensor along the shortest W=O bond. These values clearly fit in the range characteristic of paramagnetic W^{6+}/W^{5+}reduced tungsten-oxo species or extended polyanions.[3,4] This resonance is likely associated to reduced tungsten-oxo surface species, as the bulk crystal structure does not contain any short W=O bonds. The second signal ESR2 located at high field exhibits a g factor (1.64) that is characteristic of W^{5+} in bulk tungsten oxides and tungsten oxide-based glasses.[5,6] Under photoexcitation with the green light (514 nm) from an Ar ion laser we observe (**Supplementary Figure 5**) an increase in intensity of the bulk W^{5+} species (ESR2) and the formation of a new ESR signal (ESR3) with g factor around

g = 2.011 and a linewidth of 30 G. This signal cannot be attributed to the formation of free oxygen-based radicals for which a *g* factor of 2.002 would be expected. The resonance can be related to the formation of electron charge carriers in the conduction band that yield the typical photocatalytic activity of mixed valence tungsten oxides.

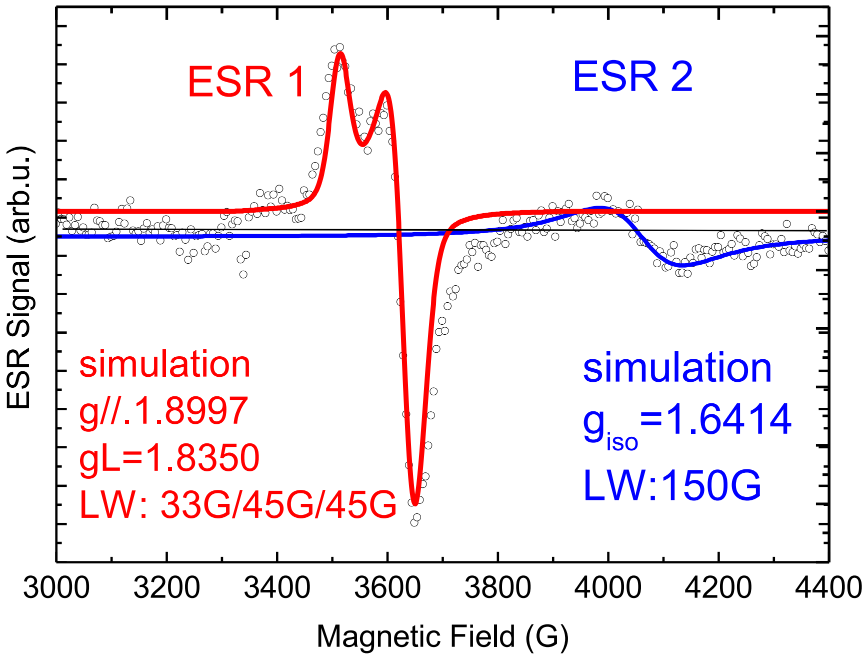

Supplementary Figure 4. X-band ESR spectrum of slightly reduced **h'-WO_3** measured at T=4 K under thermal equilibrium.

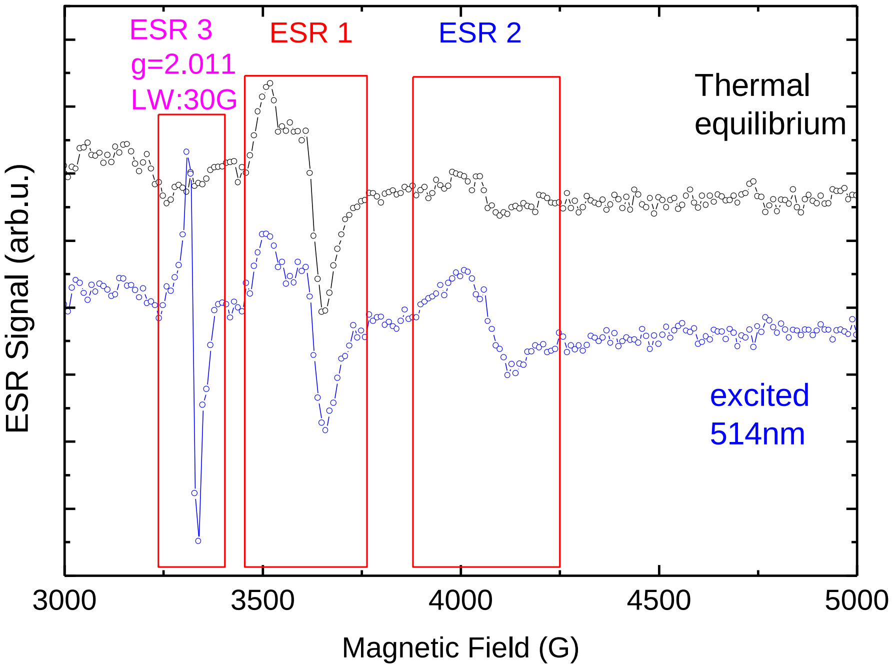

Supplementary Figure 5. X-band ESR spectra of slightly reduced **h'-WO_3** measured at T=4 K before and under photoexcitation (514 nm).

h'-$H_{0.07}WO_3$ bronze. The proton bronze **h'-$H_{0.07}WO_3$** was also studied by ESR and FMR (**Supplementary Figures 6** and **7**). At room temperature the sample shows only a >1000 G broad high intensity X-band ESR signal (**Supplementary Figure 6**) that, according to its width, intensity and low resonance field (735 G), cannot be attributed to a paramagnetic defect. At Q-band (33 GHz), the lineshape is better resolved and can be simulated with an anisotropic Lorentz lineshape. The anisotropy is due to the particle shape induced-magnetic anisotropy and its high value as compared to the resonance field. When measured in the temperature range 300 K to 4 K, the intensity of this signal is independent of the temperature in the whole range (**Supplementary Figure 7**). Further, the spectrum broadens slightly and shifts to lower resonance field with decreasing temperature. All these features are the fingerprint of a ferro/ferri-magnetic material. We thus attribute the origin of this wide signal to a ferro/ferri-magnetic phase. The small changes in its characteristics between 4 K and 300 K indicate that the blocking temperature should be above room temperature. The origin of this ferro/ferri-magnetic phase is ascribed to a carrier-mediated FM interaction between the spin S=1/2 W^{5+} centers at relatively high concentration. Contrary to previously mentioned individual W^{5+}centers observed at a *g*-factor of 1.6, the effective resonant field in the ferromagnetic phase is shifted to lower fields. In a ferromagnetic system the internal magnetic field is the sum of the Zeeman field, the dipolar interaction related component and the exchange interaction related contribution. The observation of a ferromagnetic phase highlights the important modifications of the magnetotransport properties that are characteristic of the formation of a mixed valence tungsten oxide bronze:

- A hopping process of the unpaired electrons occurs between W^{5+} and W^{6+} ions *via* thermal activation coupled to phonons. Such hopping yields an increase in the conductivity of the bronze by intervalence transitions.

- The strong line broadening is related to the ferro/ferri-magnetic phase and also to the magnetic anisotropies in the nanosheets with high anisotropy factor.

Finally, under photoexcitation at T = 4 K with the light from the HBO lamp, we observe an additional spectrum at g = 2.004 and a linewidth of 20 G (**Supplementary Figure 7**). This signal is generally attributed to oxygen vacancy-related defects. Note also that minor amounts of Mo^{4+} are detected at a typical *g* of 4.3 (**Supplementary Figures 7** and **8**), which can arise from the usual contamination of the tungsten precursor, sodium tungstate dihydrate, by molybdenum species.

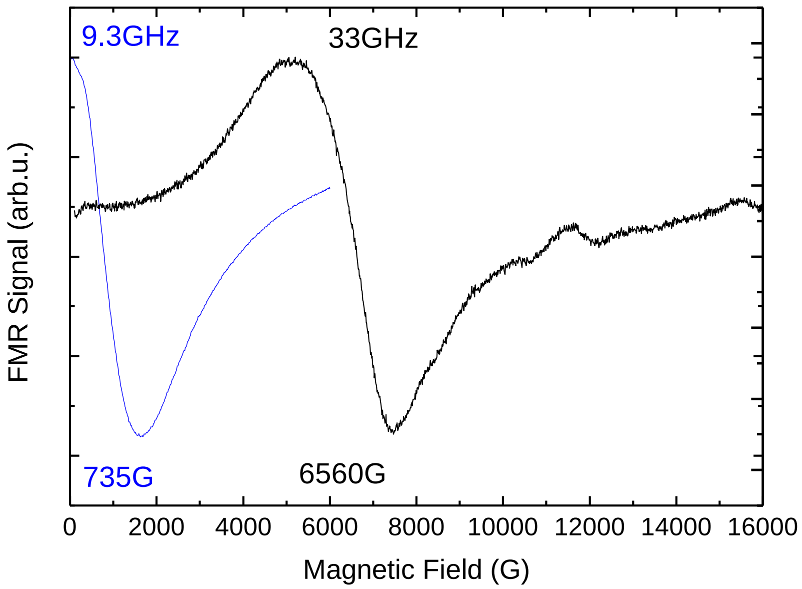

Supplementary Figure 6. Room temperature X-band (blue) and Q-band (black) FMR spectra of the proton bronze **h'-$H_{0.07}WO_3$**.

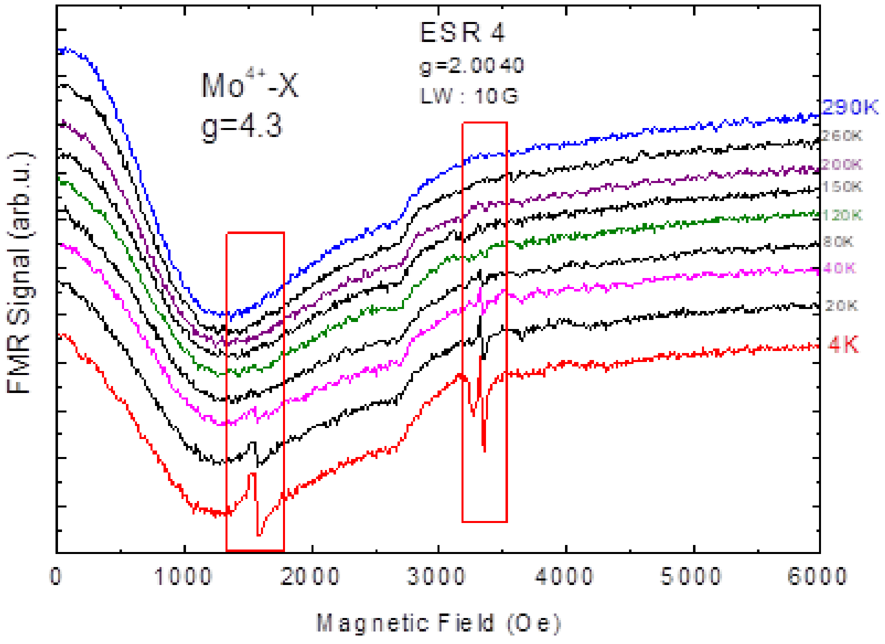

Supplementary Figure 7. X-band FMR/ESR spectra of the proton bronze **h'-$H_{0.07}WO_3$** as a function of temperature under HBO photoexcitation.

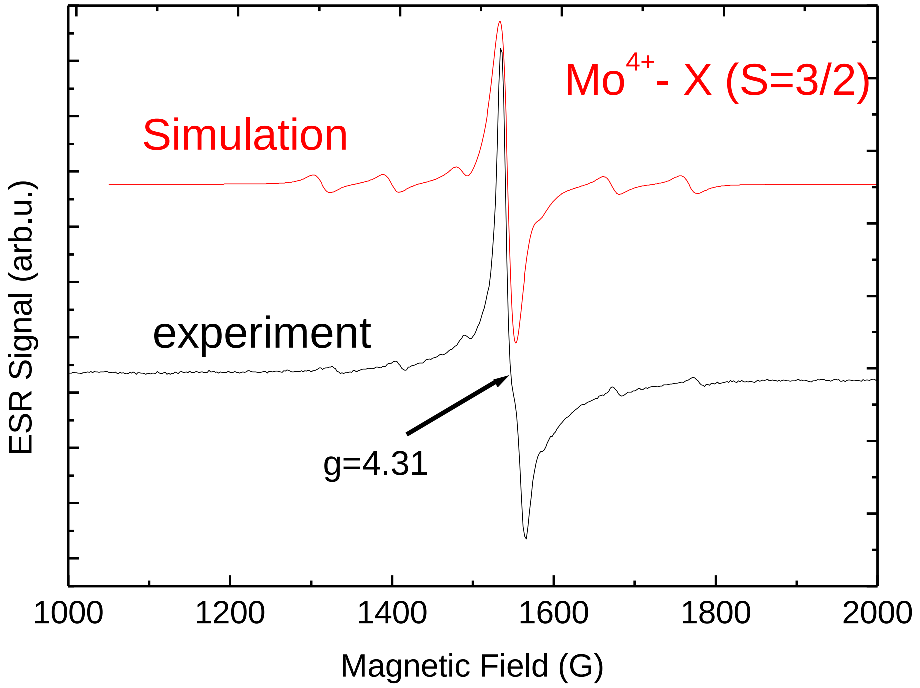

Supplementary Figure 8. ESR spectrum at 4 K and simulation of a Mo^{4+}-X center in the proton bronze **h’-$H_{0.07}WO_3$**.

Composition of h'-WO_3 and its hydrogen bronze

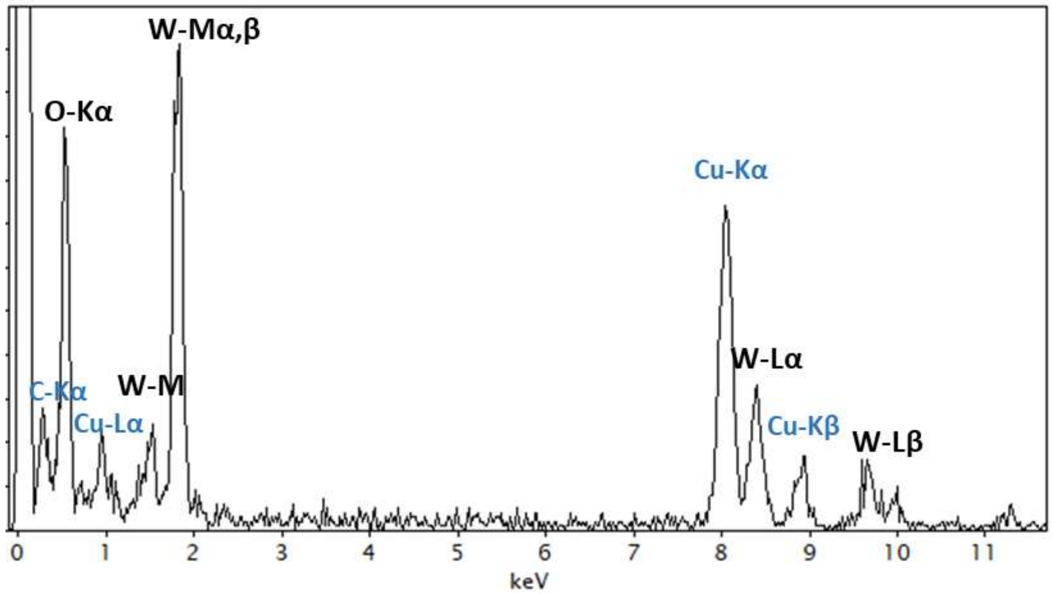

Supplementary Figure 9. EDS spectrum of the **h'** bronze. No characteristic peak of sodium and nitrogen could be detected.

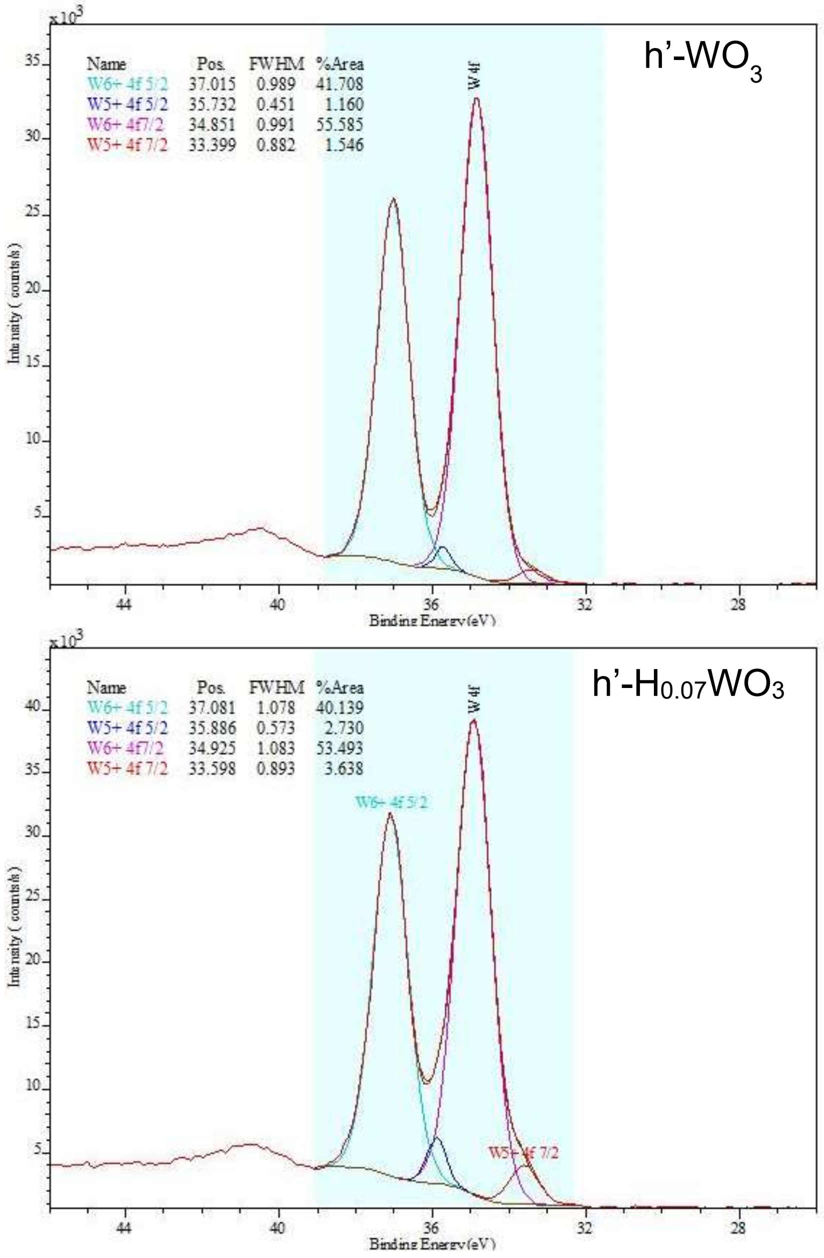

Supplementary Figure 10. W4f XPS peaks and corresponding deconvolutions for the **h'** bronze and **h'-WO_3**. W^{5+} residuals (ca. 3% of the total amount of tungsten) are observed in oxidized **h'-WO_3** and are due to partial reduction under the X-ray irradiation in the ultrahigh vacuum chamber of the XPS apparatus. This gives a rough evaluation of the uncertainty of the measurement. The amount of W^{5+} in the reduced proton bronze of **h'-WO_3** is about 7% of the total amount of tungsten. The resulting negative charge of the framework is compensated by protons. This results in the bronze formula: **h'-$H_{0.07 \pm 0.03}WO_3$** , consistent with ion exchange data (see main text).

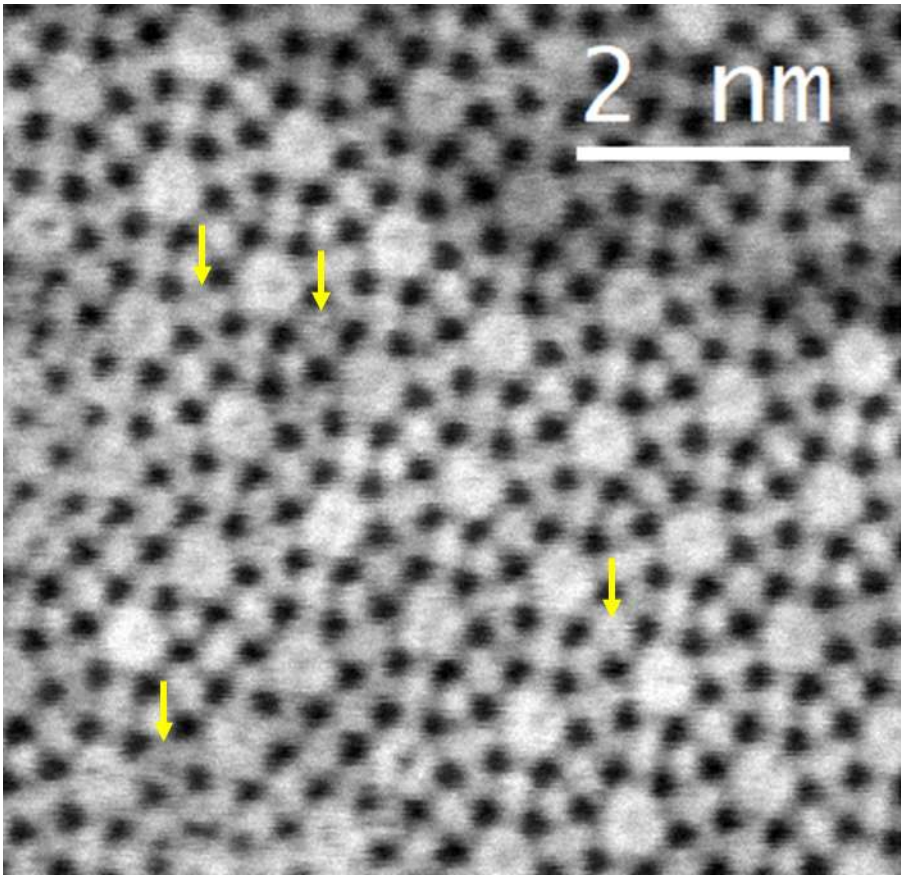

Supplementary Figure 11. STEM-ABF image of the **h’-$H_{0.07}WO_3$** bronze showing dark contrast in all $(WO_6)_6$ channels and in some of the $(WO_6)_4$ channels (arrows). The dark contrast spots are not observed in STEM-HAADF mode and thus highlight the presence of a light element: hydrogen.

Modeling

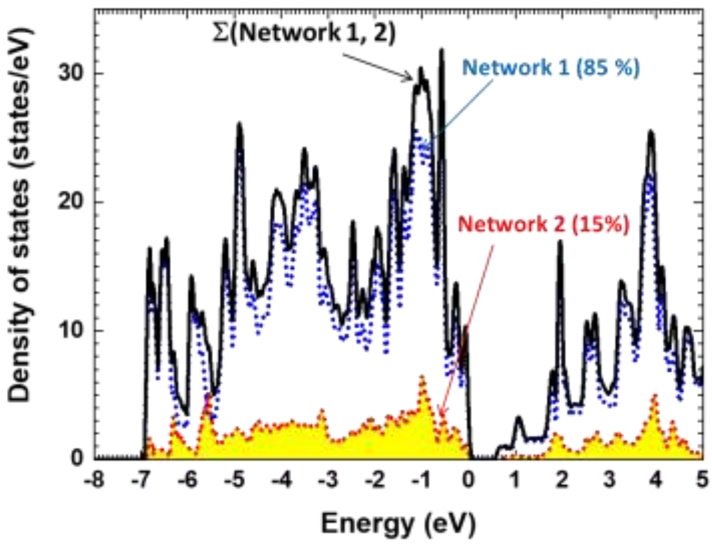

Supplementary Figure 12. Total DOS of the oxidized sample calculated from the contributions of the **h'** and **rotated h'** networks evaluated from XRD-based structure refinement.

If standard DFT methods underestimate bang gap values, it is rather pronounced in the case of hexagonal-WO_3 as reported elsewhere[7] with gap ~ 0.5 eV using the PBE method. However, a qualitative analysis and comparison within PBE is relevant. We have to mention that reports have focused on testing a variety of schemes and in particular using hybrid functionals,[8] for instance, for the γ-monoclinic phase, 3.16 eV is reported with the HSE06 functional, while our PBE method led to 1.22 eV consistently with previous PBE studies with values in the average range ~1.1-1.45 eV.[9]

To model hydrogen insertion in the cavities, the two following models were calculated and converged after DFT full optimization:

- Hydrogen atoms located at the center of the hexagonal channels (**Supplementary Figure 12**) with a 2*a**2*b***c* supercell yield an overall composition of $H_{0.125}WO_3$ close to the experimental one. Standard spin-polarized calculations yield DOS similar to **h'-WO_3** but with additional states corresponding to the non-ionized state H *1s* in the region corresponding to the band gap of **h'-WO_3** with the top of this additional state being crossed by the Fermi level

(**Supplementary Figure 12**). Such features have been reported in computational studies on **h-WO$_3$** with similar incorporation of hydrogen in the cavities.[7] This picture preserves W^{6+} states which do not allow a polaron mechanism involving lower oxidation states of W that are expected to explain the coloration.

- Hydrogen located at the edge of the $(WO_6)_6$ channels in the vicinity of an oxygen atom **(Supplementary Figure 13)** using the supercell *a***b**2*c* led to a composition of $H_{0.083}WO_3$ in agreement with the experiment. This geometry leads to the formation of OH groups. A reasonable structural model converged with O-H distance ~0.975 Å. Compared to the previous model, the H (s) states are lying much lower in the valence band, in agreement with results from other metal hydroxides.[10]

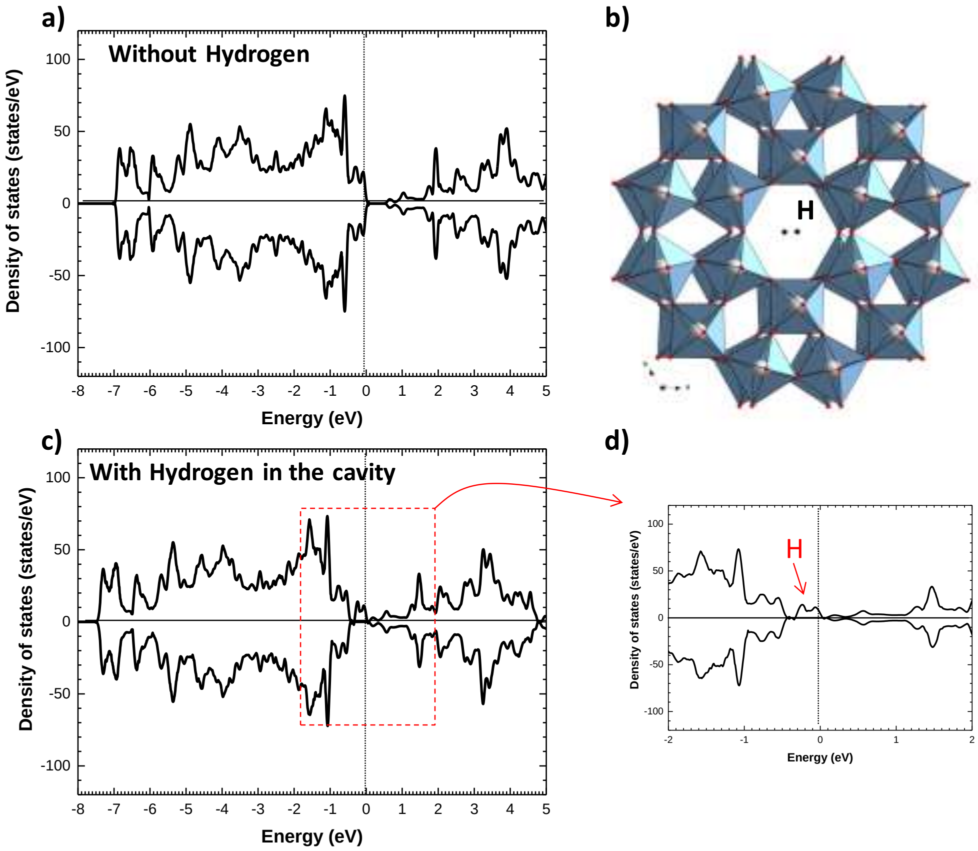

Supplementary Figure 13. (**a**) Total DOS of the oxide supercell 2*a**2*b***c*. (**b**) Structure of the supercell h'-$H_{0.125}WO_3$ with hydrogen in the center of the hexagonal cavities. (**c**) Total DOS of the supercell with hydrogen in the cavities. (**d**) Zoom of the region of the DOS with the H contribution, H states are pointed. A plane wave cutoff energy of 550 eV and 24 (44) *k* points in the irreducible Brillouin zone were used for the relaxation (DOS). The Fermi level is set to 0.

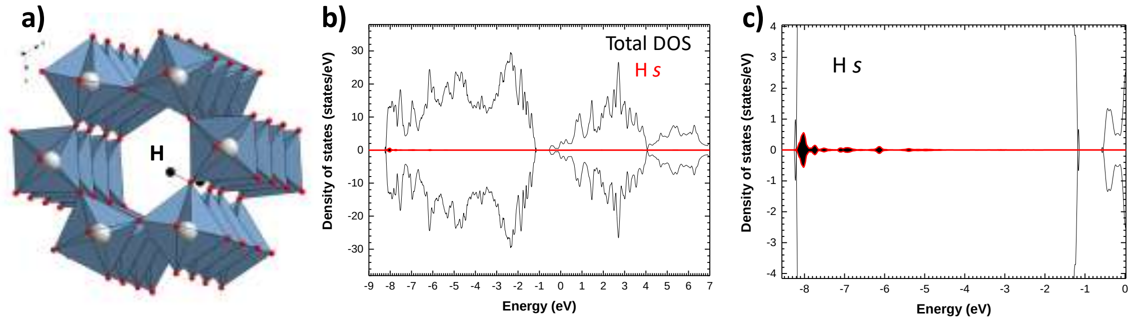

Supplementary Figure 14. (**a**) Structure of the supercell h'-$H_{0.083}WO_3$ with hydrogen in a hexagonal cavity and in the vicinity of an oxygen atom. (**b**) Corresponding Total DOS. (**c**) Zoom of the region of the DOS with the contribution of the H atom of the supercell, H states are highlighted with red contour and black filling. A plane wave energy cutoff of 550 eV and 21 (65) *k* points in the irreducible Brillouin zone were used for the relaxation (DOS). The Fermi level is set to 0.

Specific surface area

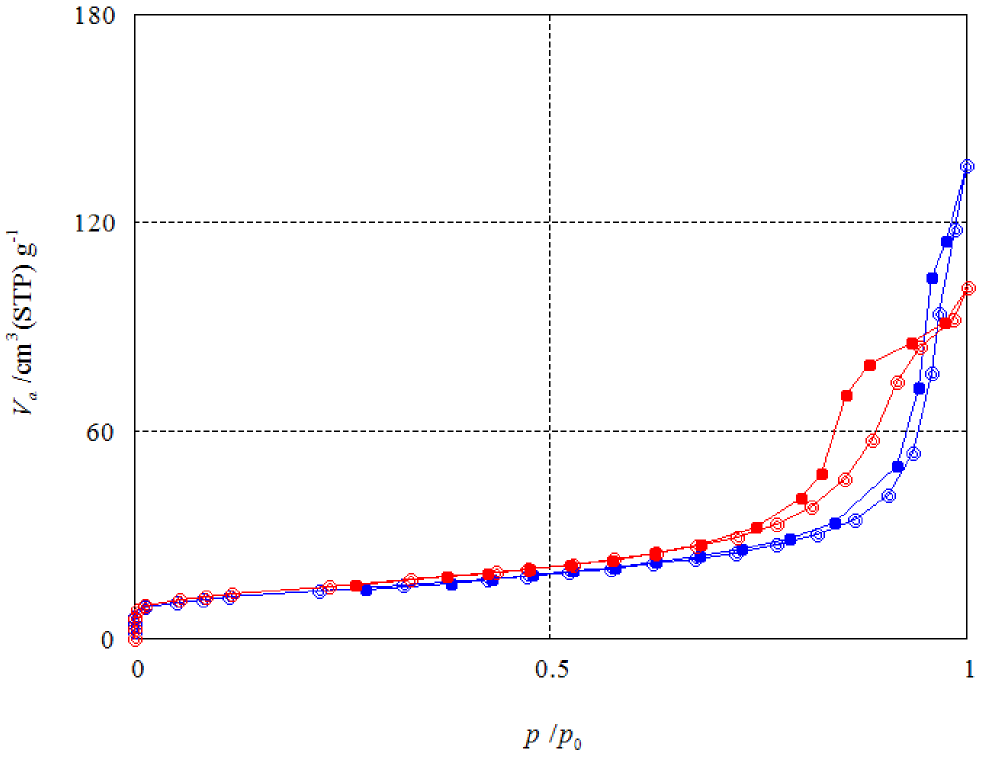

Supplementary Figure 15. N_2 sorption isotherms of **h'-WO_3** (red) and **h-WO_3** (blue). For **h'-WO_3**, the hysteresis at ~0.8 relative pressure indicates some mesopores with a mean diameter of ~12 nm according to the BJH analysis. They are ascribed to inter-platelet spaces. At low relative pressure, the steep increase in adsorbed volume indicate microporosity, ascribed to the crystal structure of both solids.

Electrochromic properties

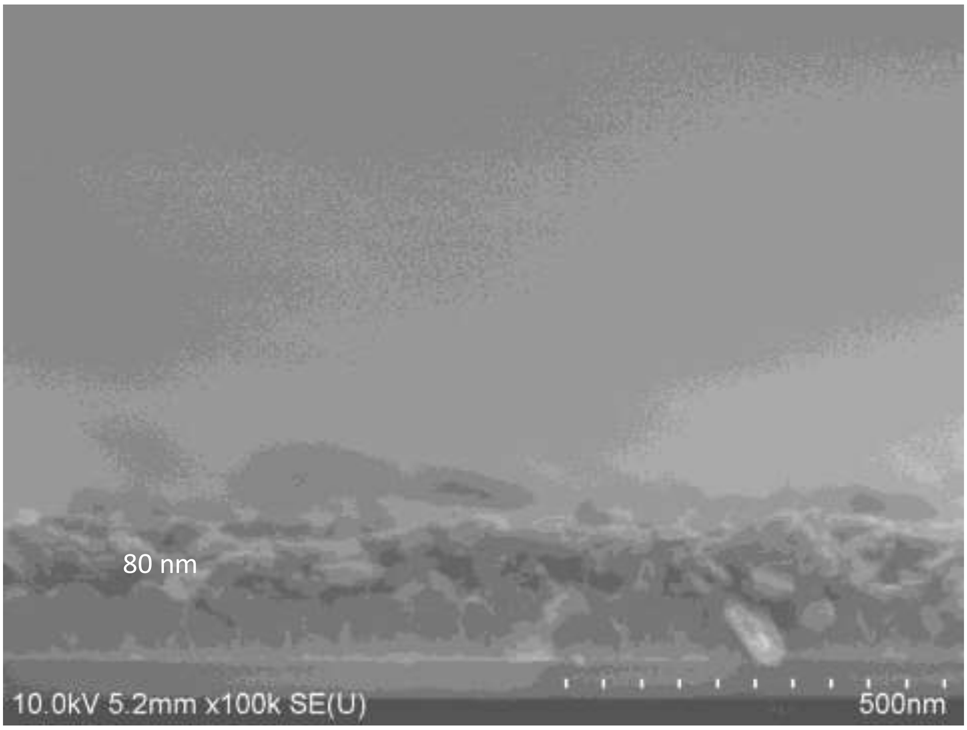

Supplementary Figure 16. SEM image showing the thickness of a **h'-WO_3**/FTO film.

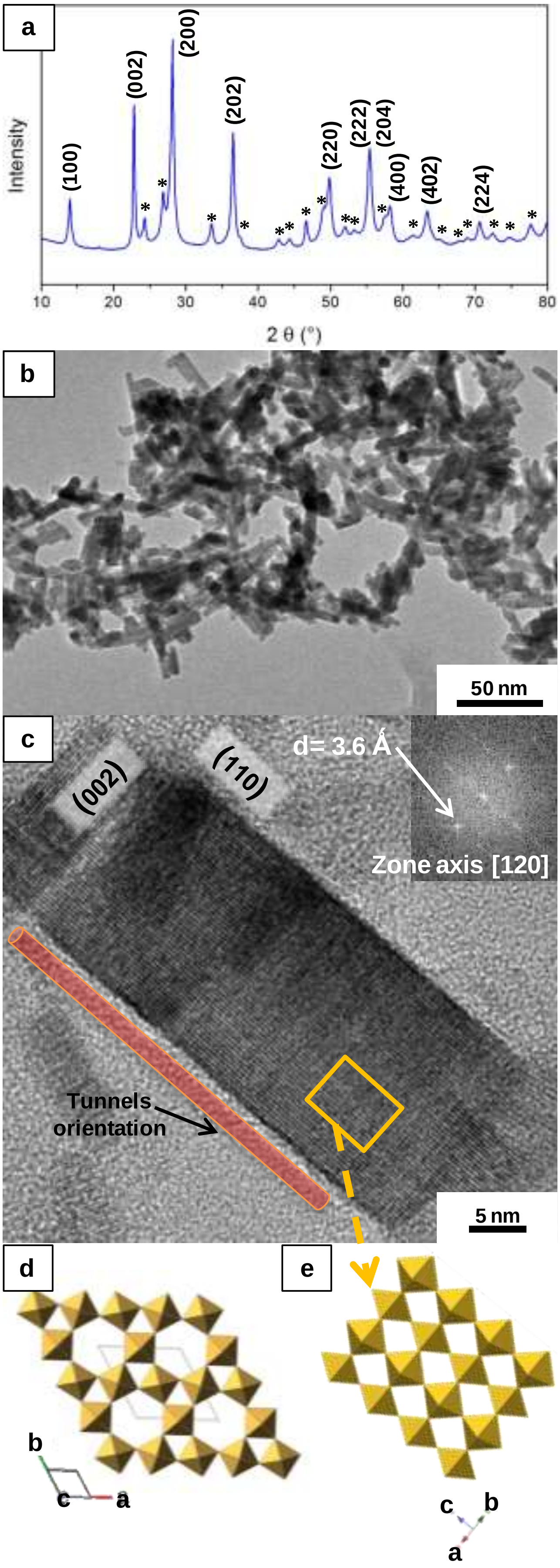

Supplementary Figure 17. h-WO_3 nanoparticles used as a comparison for h'-WO_3 electrochromic films. (**a**) Powder XRD pattern (indexes and stars: ICDD 04-007-0979 reference), (**b**) TEM and (**c**) HRTEM images showing the (**d, e**) crystal orientation of the **h-WO_3** nanorods. **h-WO_3** was synthesized with a protocol close to the one used for **h'-$H_{0.07}WO_3$**: sodium tungstate dihydrate, $Na_2WO_4 \cdot 2H_2O$ (Sigma) was dissolved in Milli-Q water to obtain a 0.15 mol L^{-1} aqueous solution. Then the pH of the solution was adjusted to 1.3 with concentrated HCl (12 mol L^{-1}). The reaction medium was heated at 120 °C in a borosilicate vial for 12 h. The resulting powder was washed by centrifugation until the supernatant pH was neutral. The sample was then dried at 40 °C under vacuum before grinding for further characterization.

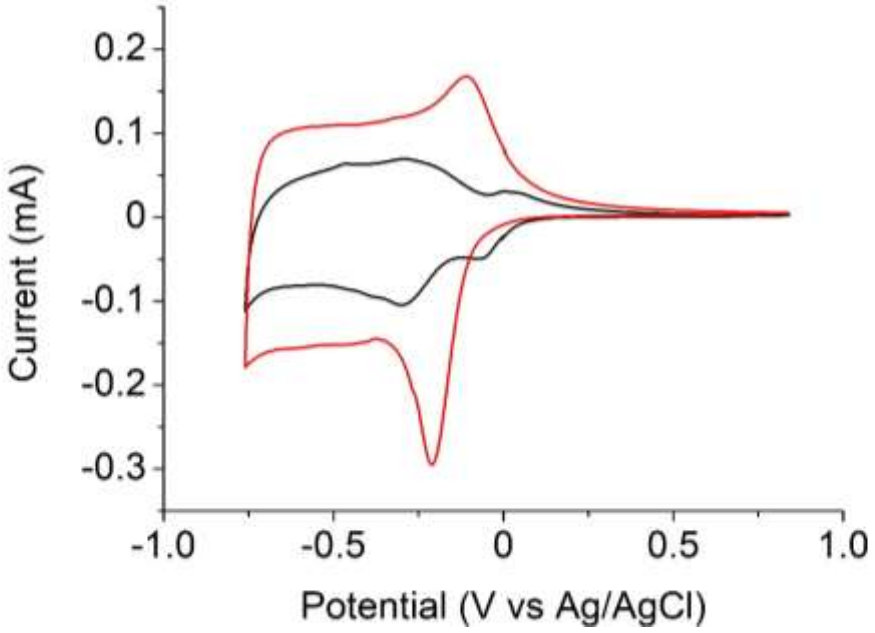

Supplementary Figure 18. Cyclic voltammograms (4th cycles) of **h'-WO_3**/FTO (red) and **h-WO_3**/FTO (black) electrodes in an H_2SO_4 0.1 mol L^{-1} aqueous electrolyte at a scan rate of 10 mV s^{-1}. Existence of one single pair of redox peaks for **h'-WO_3**, *versus* two pairs for **h-WO_3**, suggests that the different proton insertion sites in the **h'** phase are energetically equivalent or that H^+ migration to these sites occurs at similar rates.

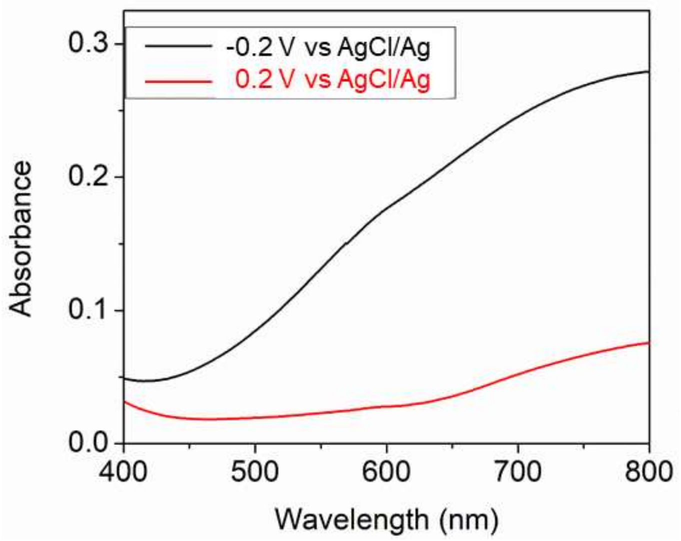

Supplementary Figure 19. UV-visible spectra of **h'-WO_3** films on FTO electrodes in the colored (-0.2 V *vs* AgCl/Ag) and bleached (-0.2 V *vs* AgCl/Ag) states.

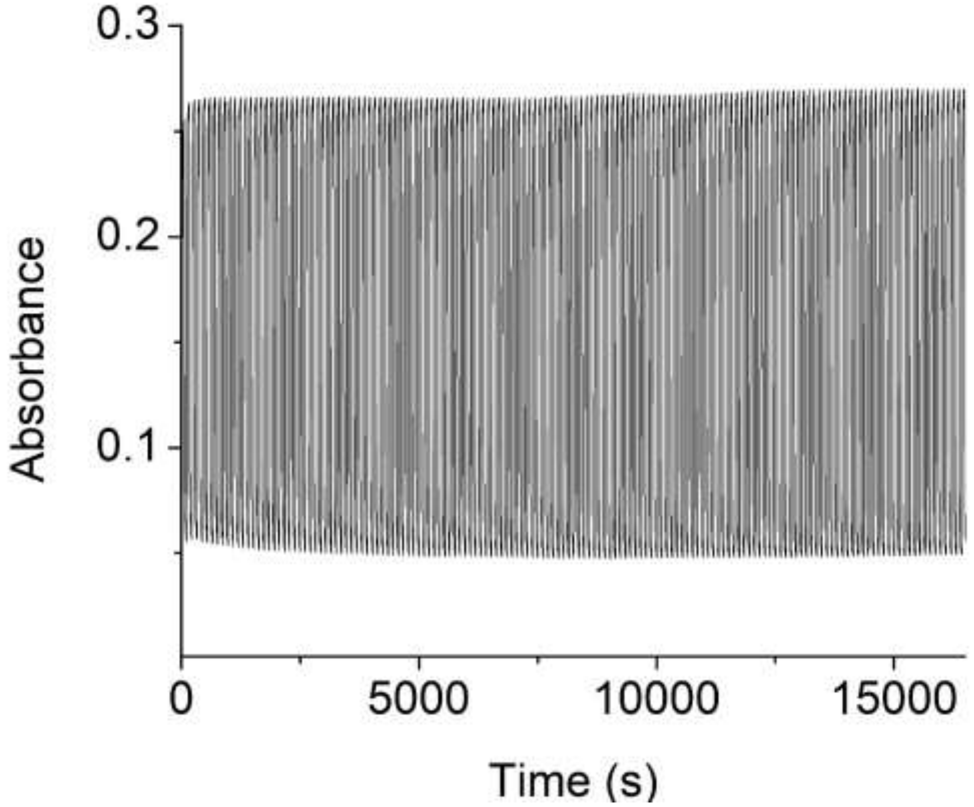

Supplementary Figure 20. Variation of the absorbance of a **h’-WO_3** film over 150 cycles between the bleached (0.2 V vs Ag/AgCl) and the colored (-0.2 V vs Ag/AgCl) states.

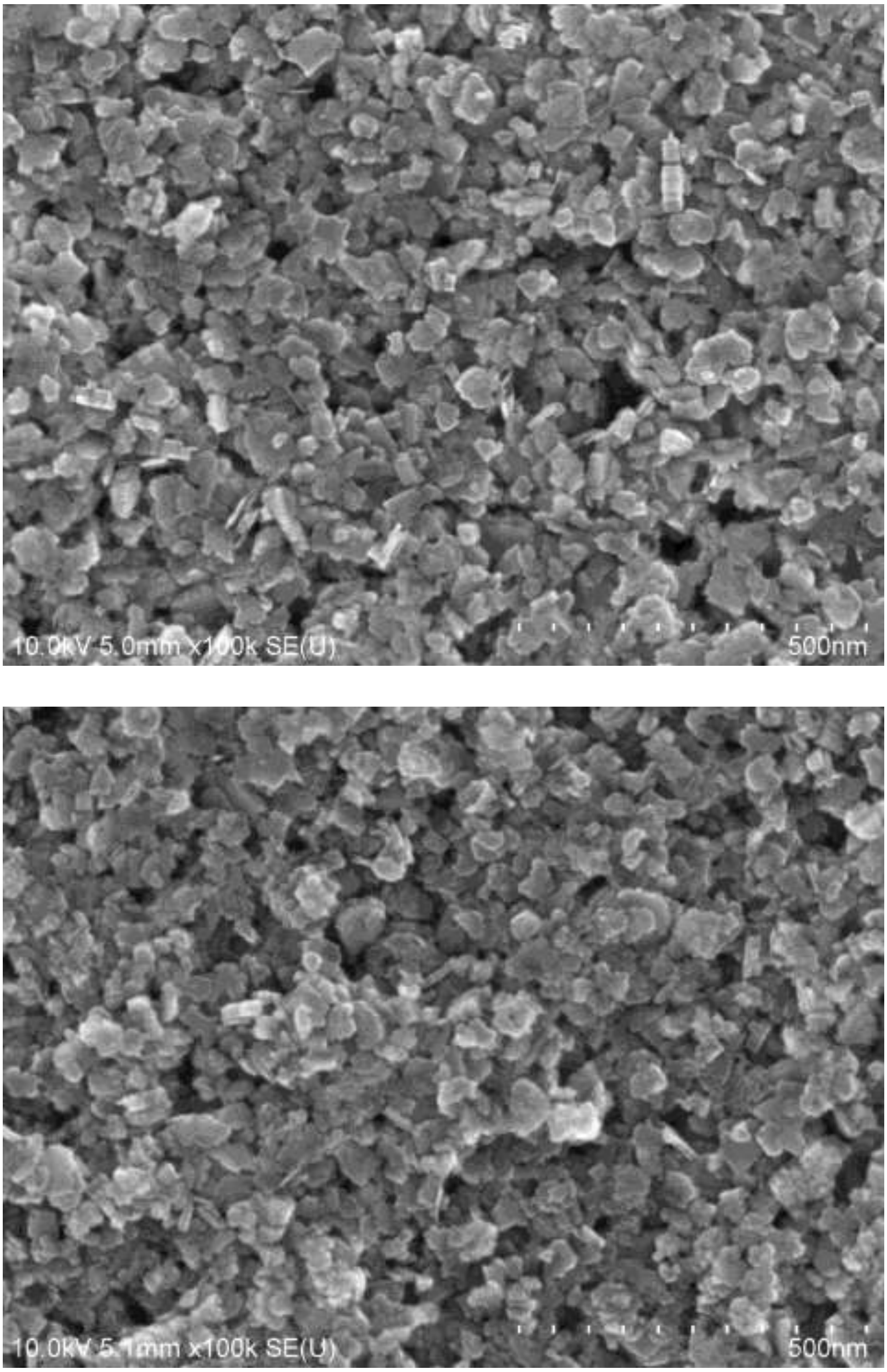

Supplementary Figure 21. SEM images of a **h’-WO_3** /FTO film in the initial state (top) and after 150 cycles (bottom) between the bleached (0.2 V vs Ag/AgCl) and the colored (-0.2 V vs Ag/AgCl) states. The film is unchanged.

Supplementary references

1. Rodriguez-Carvajal, J. FULLPROF.2k : Rietveld, profile matching and integrated intensity refinement of X-Ray and neutron data. (2001).
2. Gerand, B., Nowogrocki, G., Guenot, J. & Figlarz, M. Structural study of a new hexagonal form of tungsten trioxide. *J. Solid State Chem.* **29,** 429–434 (1979).
3. Sanchez, C., Livage, J., Launay, J. P. & Fournier, M. Electron Delocalization in Mixed-Valence Tungsten Polyanions. *J. Am. Chem. Soc.* **105,** 6817–6823 (1983).
4. Poulos, A. S. *et al.* Photochromic Hybrid organic−inorganic liquid-crystalline materials built from nonionic surfactants and polyoxometalates: Elaboration and structural study. *Langmuir* **24,** 6285–6291 (2008).
5. Schirmer, O. F. & Salje, E. The W^{5+} polaron in crystalline low temperature WO_3 ESR and optical absorption. *Solid State Commun.* **33,** 333–336 (1980).
6. Pifer, J. H. & Sichel, E. K. Electron resonance study of hydrogen-containing WO_3 films. *J. Electron. Mater.* **9,** 129–140 (1980).
7. Ingham, B., Hendy, S. C., Chong, S. V. & Tallon, J. L. Density-functional studies of tungsten trioxide, tungsten bronzes, and related systems. *Phys. Rev. B - Condens. Matter Mater. Phys.* **72,** 3–6 (2005).
8. Gerosa, M. *et al.* Electronic structure and phase stability of oxide semiconductors: Performance of dielectric-dependent hybrid functional DFT, benchmarked against G W band structure calculations and experiments. *Phys. Rev. B* **91,** 155201 (2015).
9. Zhang, T. *et al.* Iron-doping-enhanced photoelectrochemical water splitting performance of nanostructured WO_3: a combined experimental and theoretical study. *Nanoscale* **7,** 2933–2940 (2015).
10. Jahangiri, S. & Mosey, N. J. Effects of reduced dimensionality on the properties of magnesium hydroxide and calcium hydroxide nanostructures. *Phys. Chem. Chem. Phys.* **19,** 1963–1974 (2017).
