# Supplementary material for: Structure and electrochromism of two-dimensional octahedral molecular sieve h’-WO3
Source: Nat Commun. 2019 Jan 18;10:327. doi: 10.1038/s41467-018-07774-x (PMC6338762; doi:10.1038/s41467-018-07774-x)
Supplement: Supplementary file 3 — Description of Additional Supplementary Files [file 41467_2018_7774_MOESM3_ESM.docx]

**Description of Supplementary Information Files**

**File Name:** Supplementary Movie 1

**Description:** Color switching of a h’-WO3 film on an FTO susbtrate, cycled between potential plateaus 0.8 and -0.8 V versus AgCl/Ag (corresponding Figure 5a and b in the main text) at 0.2 Hz.

**File Name:** Supplementary Data 1

**Description:** Crystallographic Information File (CIF) of h’-WO3

**File Name**: Supplementary Data 2

**Description:** CheckCIF file for h’-WO3
