# Supplementary material for: Structure and electrochromism of two-dimensional octahedral molecular sieve h’-WO3
Source: Nat Commun. 2019 Jan 18;10:327. doi: 10.1038/s41467-018-07774-x (PMC6338762; doi:10.1038/s41467-018-07774-x)
Supplement: Supplementary file 6 — Supplementary Data 2 [file 41467_2018_7774_MOESM6_ESM.pdf]

checkCIF (basic structural check) running

Checking for embedded fcf data in CIF ...
No extractable fcf data in found in CIF

checkCIF/PLATON (basic structural check)

You have not supplied any structure factors. As a result the full set of tests cannot be run.

THIS REPORT IS FOR GUIDANCE ONLY. IF USED AS PART OF A REVIEW PROCEDURE FOR PUBLICATION, IT SHOULD NOT REPLACE THE EXPERTISE OF AN EXPERIENCED CRYSTALLOGRAPHIC REFEREE.

You have not supplied any structure factors. As a result the full set of tests cannot be run.

```
No syntax errors found.                        CIF dictionary
Please wait while processing ....              Interpreting this report
```

Datablock: I

```
Bond precision:      = 0.0000 A                    Wavelength=1.54180
Cell:          a=9.9975(9)      b=9.9975(9)      c=3.9199(6)
               alpha=90         beta=90          gamma=120
Temperature: 293 K
                    Calculated                        Reported
Volume              339.30(9)                         339.30(9)
Space group         P 6/m m m                         P 6/m m m
Hall group          -P 6 2                            -P 6 2
Moiety formula      0.857(O18 W6), 0.143(O18 W6)      0.857(O18 W6), 0.143(O18 W6)
Sum formula         O18 W6                            O18 W6
Mr                  1391.04                           1391.04
Dx,g cm-3           6.808                             6.808
Z                   1                                 1
Mu (mm-1)           92.329                            92.329
F000                588.0                             588.0
F000'               556.79
h,k,lmax            12,12,4                           12,12,4
Nref                161                               161
Tmin,Tmax
Tmin'
Correction method= Not given
Data completeness= 1.000        Theta(max)= 69.997
R(reflections)=                    wR2(reflections)=
S =                   Npar=
```

```
The following ALERTS were generated. Each ALERT has the format
       test-name_ALERT_alert-type_alert-level.
Click on the hyperlinks for more details of the test.
```

Alert level B

```
PLAT213_ALERT_2_B Atom W          has ADP max/min Ratio .....       5.0 prolat
PLAT213_ALERT_2_B Atom W'         has ADP max/min Ratio .....       5.0 prolat
```

Alert level G

```
PLAT004_ALERT_5_G Polymeric Structure Found with Maximum Dimension          3 Info
PLAT301_ALERT_3_G Main Residue  Disorder ..............(Resd  1  )       100% Note
PLAT301_ALERT_3_G Main Residue  Disorder ..............(Resd  2  )       100% Note
PLAT860_ALERT_3_G Number of Least-Squares Restraints .............          3 Note
PLAT982_ALERT_1_G The  O-f'=       0.047 Deviates from the  IT-value      0.049   Ch
PLAT982_ALERT_1_G The  W-f'=      -6.048 Deviates from the  IT-value     -5.473   Ch
PLAT983_ALERT_1_G The  W-f"=       5.577 Deviates from the  IT-Value      5.577   Ch
```

```
   0 ALERT level A = Most likely a serious problem - resolve or explain
   2 ALERT level B = A potentially serious problem, consider carefully
   0 ALERT level C = Check. Ensure it is not caused by an omission or oversight
   7 ALERT level G = General information/check it is not something unexpected

   3 ALERT type 1 CIF construction/syntax error, inconsistent or missing data
   2 ALERT type 2 Indicator that the structure model may be wrong or deficient
   3 ALERT type 3 Indicator that the structure quality may be low
   0 ALERT type 4 Improvement, methodology, query or suggestion
   1 ALERT type 5 Informative message, check
```

It is advisable to attempt to resolve as many as possible of the alerts in all categories. Often the minor alerts point to easily fixed oversights, errors and omissions in your CIF or refinement strategy, so attention to these fine details can be worthwhile. In order to resolve some of the more serious problems it may be necessary to carry out additional measurements or structure refinements. However, the purpose of your study may justify the reported deviations and the more serious of these should normally be commented upon in the discussion or experimental section of a paper or in the "special_details" fields of the CIF. checkCIF was carefully designed to identify outliers and unusual parameters, but every test has its limitations and alerts that are not important in a particular case may appear. Conversely, the absence of alerts does not guarantee there are no aspects of the results needing attention. It is up to the individual to critically assess their own results and, if necessary, seek expert advice.

Publication of your CIF in IUCr journals

A basic structural check has been run on your CIF. These basic checks will be run on all CIFs submitted for publication in IUCr journals (*Acta Crystallographica*, *Journal of Applied Crystallography*, *Journal of Synchrotron Radiation*); however, if you intend to submit to *Acta Crystallographica Section C* or *E* or *IUCrData*, you should make sure that full publication checks are run on the final version of your CIF prior to submission.

Publication of your CIF in other journals

Please refer to the *Notes for Authors* of the relevant journal for any special instructions relating to CIF submission.

PLATON version of 30/01/2018; check.def file version of 30/01/2018

Datablock I - ellipsoid plot

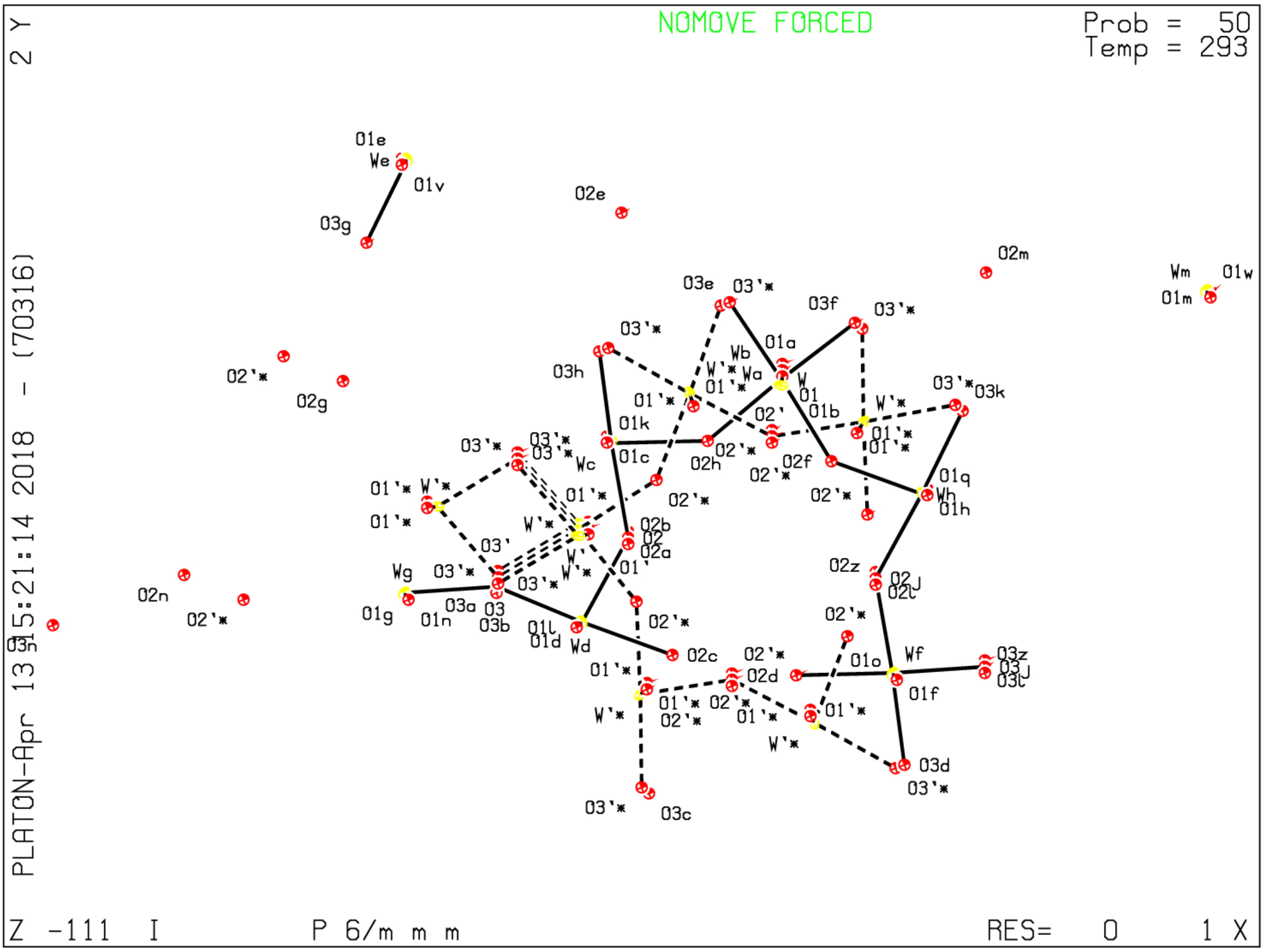

[Download CIF editor (publCIF) from the IUCr](#)
[Download CIF editor (enCIFer) from the CCDC](#)
[Test a new CIF entry](#)
